# Tauroursodeoxycholic Acid Induces Liver Regeneration and Alleviates Fibrosis Through GATA3 Activation

**DOI:** 10.3390/biomedicines13040910

**Published:** 2025-04-09

**Authors:** Chongyang Bai, Xiaojing Song, Jiexi Yan, Jun Xu, Yongqiang Zhou, Zongbin Sun, Qiuxia Zheng, Yue Zhang, Ruixi Chen, Xiaoyi Jin, Yi Shao, Yande Xie, Lele Yang, Fupeng Zhong, Yuting Zhang, Jiatai Li, Runfeng Li, Shaolin Yan, Xun Li

**Affiliations:** 1 The First School of Clinical Medicine, Lanzhou University, Lanzhou 730000, China; 2 Department of General Surgery, The First Hospital of Lanzhou University, Lanzhou 730000, China; 3 National Clinical Key Specialty of General Surgery, The First Hospital of Lanzhou University, Lanzhou 730000, China; 4 Hepatopancreatobiliary Surgery Institute of Gansu Province, Lanzhou 730000, China; 5 Clinical Research Center for General Surgery of Gansu Province, Lanzhou 730000, China; 6 Precision Medicine Laboratory, The First Hospital of Lanzhou University, Lanzhou 730000, China; 7 Department of Vascular Surgery, The First Hospital of Lanzhou University, Lanzhou 730000, China; 8 Cancer Prevention and Treatment Center of Lanzhou University School of Medicine, Lanzhou 730000, China; 9 Key Laboratory Biotherapy and Regenerative Medicine of Gansu Province, Lanzhou 730000, China

**Keywords:** Tauroursodeoxycholic acid, liver regeneration, liver fibrosis, GATA3

## Abstract

**Background**: Liver regeneration is a critical measure of liver health and plays an essential role in inhibiting the progression of fibrotic lesions and preventing liver failure after hepatocellular carcinoma surgery. However, there are no approved drugs to address this clinical challenge. **Methods**: The effects of TUDCA on liver regeneration and fibrosis were studied using BRL-3A cells, a partial hepatectomy (PH) rat liver regeneration model, and a carbon tetrachloride (CCl_4_)-induced liver fibrosis model. GATA3-knockdown BRL-3A cells were employed to assess the role of GATA3 in TUDCA-induced proliferation. **Results**: TUDCA promoted the proliferation of BRL-3A cells and enhanced liver regeneration in PH rats while ameliorating liver fibrosis in CCl_4_-treated rats. Additionally, the knockdown of GATA3 eliminated the proliferative effect of TUDCA on BRL-3A cells. **Conclusions**: TUDCA promotes liver regeneration and alleviates liver fibrosis by activating GATA3.

## 1. Introduction

In China, more than half of the population suffers from varying degrees of chronic liver diseases, including fatty liver disease, liver fibrosis, and cirrhosis [1]. Mature hepatocytes have limited self-renewal capacity and generally do not undergo cell division. However, during acute liver injury, these cells exhibit remarkable regenerative potential. In contrast, prolonged chronic liver injury impairs hepatocyte function, leading to cirrhosis and potentially hepatocellular carcinoma (HCC). Additionally, for HCC patients, insufficient residual liver volume after tumor resection can result in inadequate liver regeneration, leading to post-hepatectomy liver failure (PHLF) [2], which carries a high mortality rate. Notably, both benign and malignant liver tumors requiring surgical resection often occur in the context of chronic liver disease. Therefore, there is an urgent need to identify effective methods to enhance natural liver recovery and repair to address liver injury.

Tauroursodeoxycholic acid (TUDCA), a water-soluble bile acid found predominantly in bear bile, has received FDA approval for managing primary biliary cirrhosis. It is synthesized in the liver through the combination of taurine with ursodeoxycholic acid (UDCA). TUDCA exerts hepatoprotective effects by mitigating oxidative stress, apoptosis, and inflammation, thereby facilitating hepatocyte recovery and regeneration. For instance, TUDCA can pharmacologically inhibit endoplasmic reticulum (ER) stress responses, overcoming NFATc1 activation and halting the progression from nonalcoholic fatty liver disease (NAFLD) to nonalcoholic steatohepatitis (NASH) [3]. In a mouse model of spinal cord injury, TUDCA alleviated secondary damage by reducing oxidative stress, apoptosis, and inflammation [4]. Moreover, TUDCA has been shown to inhibit ER stress-mediated hepatocyte apoptosis, thus, exhibiting protective effects in models of liver injury [5,6]. Transcription factors of the GATA family hold significant importance in the differentiation and specialization of cell lineages. GATA3 deletion has been associated with severe abnormalities in multiple organs, including brain and spinal cord malformations and impaired intrahepatic hematopoiesis, in addition to defects in bone marrow-derived hematopoietic cell lineages [7]. Furthermore, emerging studies indicate that GATA3 serves as an important molecular marker and transcription factor involved in the development of various cancers, including breast cancer [8,9], bladder cancer [10], T-cell lymphoma [11], cervical cancer, prostate cancer, and lung cancer [12]. Collectively, these findings suggest that the role of GATA3 extends beyond hematopoietic cells and is significantly underestimated in other organs. Additionally, research by Pandolfi et al. [7] highlighted the critical role of GATA3 in neuronal cell differentiation, supporting the hypothesis that GATA3 may regulate liver cell differentiation and mediate the therapeutic effects of TUDCA on liver regeneration through transcriptional regulation.

Despite the hepatoprotective properties of TUDCA, current studies lack evidence that TUDCA directly promotes hepatocyte proliferation. TUDCA’s primary mechanism of action involves its antioxidative and antiapoptotic properties, which create a favorable environment for hepatocyte survival, indirectly supporting the liver regeneration process. It remains uncertain whether TUDCA directly influences hepatocyte regeneration, and the mechanisms involved have yet to be fully understood. This research focused on analyzing how TUDCA contributes to both tissue regeneration and the suppression of fibrosis, while examining the involvement of GATA3 in these processes.

## 2. Materials and Methods

### 2.1. Cell Culture

BRL-3A cells, obtained from Cell Bank/Stem Cell Bank, Chinese Academy of Sciences, were cultured in DMEM (Gibco, Grand Island, NY, USA) supplemented with 10% (*v*/*v*) FBS (HyClone, South Logan, UT, USA). The culturing process was conducted under tightly regulated conditions, maintaining a temperature of 37 °C and a 5% CO_2_ atmosphere to ensure optimal cell growth.

### 2.2. CCK-8 Proliferation Assay

BRL-3A cells were enzymatically digested and then plated in 96-well plates at a concentration of 3000 cells per well. Each experimental group included five replicates to ensure reliability and accuracy of the results. On the following day, the experimental groups were treated with TUDCA solutions (Bruschettini S.R.L., Genova, Liguria, Italy) at various concentrations (1.5625 μM–400 μM) and incubated for 24 h. Afterward, 10 μL of CCK-8 reagent was introduced into each well and allowed to incubate until the color change occurred. Absorbance at 450 nm was determined using an enzyme marker, with the results documented and evaluated.

### 2.3. Cell Cycle Analysis

BRL-3A cells were enzymatically treated and distributed into 6-well plates, ensuring three replicates were included for each experimental group. On the following day, experimental groups were treated with TUDCA (3.125 μM), while control groups received an equal volume of PBS. Following a 24 h incubation, the treated cells were collected and preserved in prechilled 75% ethanol, then kept at 4 °C overnight. The fixed cells were subsequently stained using a cell cycle detection kit (Elabscience Biotechnology Co., Ltd., Wuhan, China) as per the manufacturer’s protocol and analyzed via flow cytometry (Agilent Technologies Inc., Santa Clara, CA, USA).

### 2.4. EdU Cell Proliferation Assay

BRL-3A cells were cultured and treated with TUDCA (3.125 µM) in the experimental group and an equal volume of PBS in the control group, with three controls in each group. 10 µM of EDU (Beyotime, Shanghai, China) was added to the culture medium, and the culture was continued for 24 h to allow the cells to be doped with EDU during cell division. At the end of the culture, the cells were rinsed with PBS and fixed with 4% paraformaldehyde for 10–15 min. After fixation, cells were soaked in an enhanced permeabilization buffer (Beyotime, Shanghai, China) for 15 min and rinsed with PBS. Click reaction labeling was performed using the Click Reaction Solution as directed in the kit instructions. Cell nuclei were subsequently stained with DAPI. The observation was performed using a fluorescence microscope.

### 2.5. Scratch Wound Healing Assay

BRL-3A cells were enzymatically processed and plated into 6-well plates at a density of 200,000 cells per well. Each experimental group included three replicates to ensure consistent and reliable data. On the following day, experimental groups were treated with TUDCA (3.125 μM) and incubated for 24 h. Scratches were made using a 1 mL pipette tip. The scratch region was monitored and evaluated at intervals of 0, 4, 8, 12, 16, and 20 h.

### 2.6. Animal Husbandry

Male Sprague Dawley (SD) rats, 8 weeks old and weighing 180–220 g, were obtained from the Animal Experimental Center of Lanzhou University(Lanzhou University, Lanzhou, China). They were housed in an SPF environment equipped with regulated ventilation and a controlled 12 h day–night cycle to maintain optimal living conditions. All procedures involving the rats strictly adhered to the relevant Laboratory Animal Management Regulations and received approval from the Ethics Committee of the First Hospital of Lanzhou University (LDYYLL-2024-52).

### 2.7. Animal Experiments

#### 2.7.1. Effects of TUDCA on Liver Regeneration After 70% Hepatectomy

To investigate the effect of TUDCA on liver regeneration after 70% partial hepatectomy (PH) in rats, SD rats were randomly divided into five groups, with five rats in each group. The random numbers for animal grouping were generated using the random function in Microsoft Excel. The experimental groups received low, medium, and high doses of TUDCA (25, 50, and 100 mg/kg/day), while the sham operation group and the model group received the control solvent. All groups were gavaged for seven days. (TUDCA was dissolved in normal saline to prepare a 10 mg/mL suspension and administered under continuous magnetic stirring to ensure uniformity.) On the seventh day, rats in the model group and TUDCA experimental groups underwent 70% PH surgery. Each group continued to receive the corresponding drug or control solvent for an additional three days. On the tenth day, the rats were executed with fasting on the day prior to the execution, and liver tissue and serum samples were collected.

#### 2.7.2. Alleviation of CCL4-Induced Liver Fibrosis by TUDCA

To evaluate the effect of TUDCA on CCl4-induced hepatic fibrosis, SD rats were randomly divided into three groups, with six rats in each group. Rats in the model and experimental groups were intraperitoneally injected with a 25% CCl4 olive oil solution at a dose of 1 mL/kg, twice a week for six consecutive weeks. In the sixth week, one rat from each group was randomly sacrificed to observe the pathological status of the liver and determine the success of the hepatic fibrosis model. Rats in the experimental group were gavaged with TUDCA (50 mg/kg/day) for 10 consecutive days starting from the seventh week, after which they were sacrificed. Rats in the control and model groups were gavaged with the same dose of control solvent for 10 days before being sacrificed. All rats were fasted the night before sacrifice. Liver tissue and serum samples were collected.

#### 2.7.3. TUDCA Promotes Liver Regeneration After Hepatectomy in the Presence of CCL4-Induced Liver Fibrosis

To evaluate the effect of TUDCA on liver regeneration after partial hepatectomy (PH) in CCl4-induced hepatic fibrosis rats, SD rats were randomly divided into three groups, with six rats in each group, and hepatic fibrosis modeling was performed according to the aforementioned method. After successful modeling, the experimental group received TUDCA at a dose of 50 mg/kg/day starting from the seventh week, while the control and model groups received the control solvent. All groups were gavaged for seven days. On the seventh day, rats in the model and experimental groups underwent PH surgery. After surgery, each group continued to receive the corresponding drug or control solvent for an additional three days. On the tenth day, the rats were executed with fasting on the day prior to the execution, and liver tissue and serum samples were collected.

### 2.8. Histological and Serum Biochemical Analysis

Liver tissues were fixed in 4% paraformaldehyde, paraffin-embedded, and deparaffinized for staining, including H&E, Masson, and Sirius Red [13]. Stained sections were analyzed using ImageJ (version 1.54f) to quantify stained areas. Serum levels of ALT and AST were assessed using commercially available kits (Nanjing Jiancheng Bioengineering Institute, Nanjing, China), in accordance with the manufacturer’s protocol.

### 2.9. Immunohistochemistry

Paraffin-embedded tissue samples were cut into slices with a thickness of 5 μm, deparaffinized in xylene, and subjected to sequential alcohol treatments for dehydration. Antigen recovery was achieved by microwave heating of the slices, followed by blocking endogenous peroxidase activity using a 3% hydrogen peroxide solution. Subsequently, the slices were left at 4 °C overnight with primary antibodies, including Ki67 (Abcam, Cambridge, MA, USA), PCNA (Proteintech, Wuhan, China), and α-SMA (Abcam, Cambridge, MA, USA). Afterward, secondary antibody incubation was performed, and the staining was visualized using a chromogenic substrate (Solarbio, Beijing, China). The results were analyzed using ImageJ software (version 1.54f).

### 2.10. Immunofluorescence

Immunofluorescence staining was carried out according to a previously established protocol [14]. Liver tissues were initially preserved in 10% formalin, buffered with PBS, and then embedded in paraffin for further processing. To detect apoptotic cells, TUNEL staining was performed using a one-step TUNEL apoptosis detection kit (Beyotime, Shanghai, China). The nuclei of the cells were subsequently stained with DAPI. Fluorescent images of the stained tissues were captured using a fluorescence microscope (Leica, Wetzlar, Germany).

### 2.11. RNA-seq and Data Analysis

Liver samples from the PH and medium-dose groups (5 samples each) were collected. RNA extraction was carried out with TRIzol reagent (Thermo Fisher, Waltham, MA, USA). The RNA samples were subsequently sequenced on the Illumina NovaSeq 6000 platform (LC Biotechnology, Hangzhou, China). Bioinformatics analysis was performed using OmicStudio (https://www.omicstudio.cn/tool, accessed on 12 December 2024), with data visualization generated through R on the same platform. The raw sequence data reported in this paper have been deposited in the Genome Sequence Archive (Genomics, Proteomics & Bioinformatics 2021) in National Genomics Data Center (Nucleic Acids Res 2022), China National Center for Bioinformation/Beijing Institute of Genomics, Chinese Academy of Sciences (GSA: CRA022037) that are publicly accessible at https://ngdc.cncb.ac.cn/gsa, accessed on 11 January 2025 [15,16].

### 2.12. RT-qPCR

RNA was extracted, and qRT-PCR was carried out following the procedure described in a previous study [17]. GAPDH was used as the reference gene for normalization. The primers employed in the qRT-PCR analysis are listed in Table S1.

### 2.13. Western Blotting

Protein extraction and Western blotting were performed according to the methods described in previous studies [17]. Anti-GATA3 antibody (Proteintech, Wuhan, China) and anti-GAPDH antibody (Proteintech, Wuhan, China) were used for primary antibodies.

### 2.14. Statistical Analysis

Data analysis and visualization were conducted with GraphPad Prism software (version 8.3). Before the analysis, a normality test was applied to the data. The t-test was used for statistical analysis between two groups, and one-way ANOVA was used for statistical analysis between three or more groups. Results are expressed as mean ± standard deviation and statistical significance was assessed at *p* < 0.05.

## 3. Results

### 3.1. TUDCA Promotes the Proliferation and Migration of BRL-3A Cells

**Figure 1:**
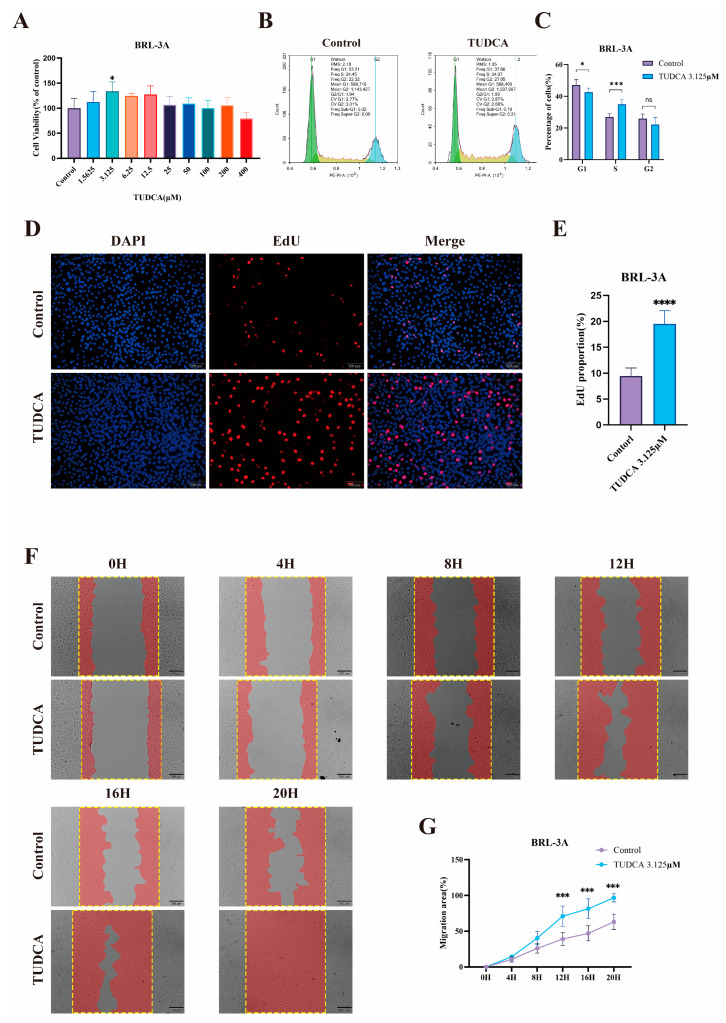
TUDCA Promotes the Proliferation and Migration of BRL-3A Cells. Statistical graph of CCK-8 assay after treatment of BRL-3A cells using TUDCA (**A**). Representative images (**B**) and statistical graphs (**C**) of cell cycle experiments after treatment of BRL-3A cells with TUDCA. Representative images (**D**) (scale bar, 100 μm) and statistical graphs (**E**) of EdU staining experiments after treatment of BRL-3A cells using TUDCA. Representative images (**F**) (scale bar, 200 μm) and statistical graphs (**G**) of scratch experiments after treatment of BRL-3A cells with TUDCA. n.s., not significant; *, *p* < 0.05; ***, *p* < 0.001; ****, *p* < 0.0001.

To validate the pro-proliferative effect of TUDCA on hepatocytes at the cellular level, we assessed the viability of BRL-3A cells treated with varying doses of TUDCA. Results indicated that higher doses of TUDCA inhibited proliferation, while a low dose (3.13 μM) promoted BRL-3A cell proliferation (Figure 1A). Experiments focusing on cell cycle progression indicated a notable rise in the number of cells undergoing proliferation following TUDCA administration (Figure 1B,C). EdU staining further confirmed this effect, as TUDCA treatment enhanced DNA replication activity, indicated by increased EdU incorporation into newly synthesized DNA (Figure 1D,E). Additionally, a scratch assay revealed TUDCA’s ability to enhance cell migration. This reflects its potential to facilitate tissue repair by enabling cells to migrate to injury sites, proliferate, and differentiate, thus, promoting tissue recovery (Figure 1F,G).

### 3.2. TUDCA Promotes Liver Regeneration in 70% Hepatectomized Rats

**Figure 2:**
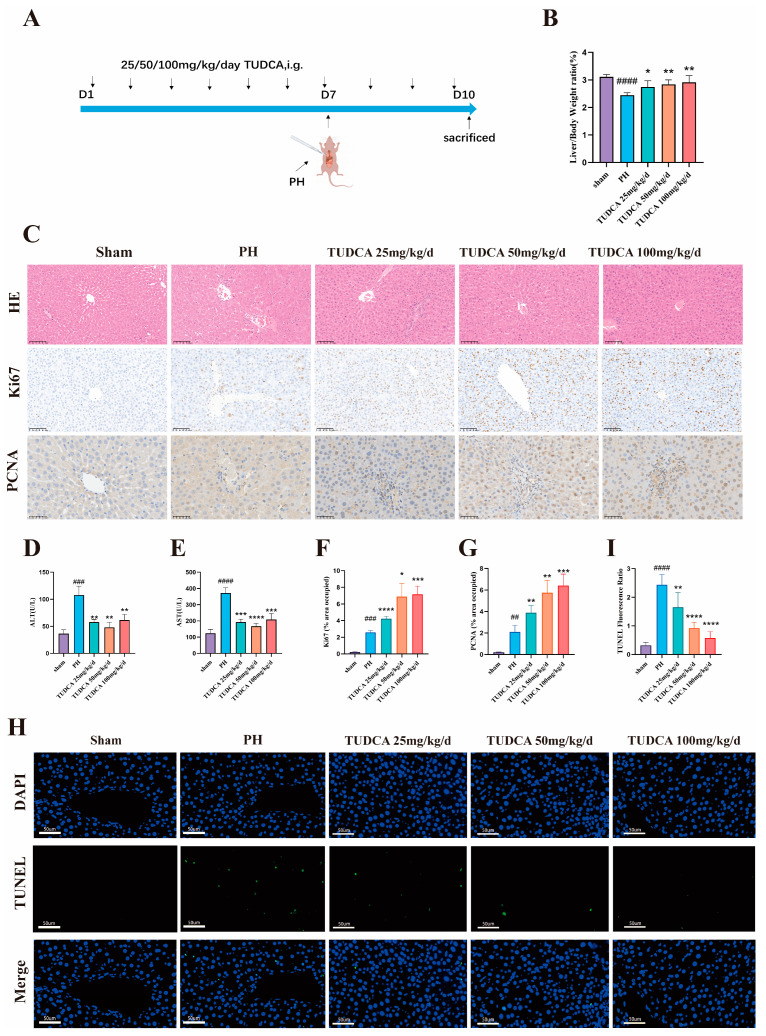
TUDCA Promotes Liver Regeneration in 70% Hepatectomized Rats. (**A**) Schematic diagram of the experimental design of TUDCA-treated 70% hepatectomised rats. (n = 5) (**B**) Liver-to-body weight ratio. (**C**,**F**,**G**) Representative and statistical images of H&E (n = 3) (scale bar, 100 μm), Ki67 (scale bar, 100 μm), and PCNA staining. (scale bar, 50 μm) (**D**,**E**) Statistical graphs of ALT and AST levels. (**H**,**I**) Representative and statistical images of TUNEL staining. (scale bar, 50 μm). ##, *p* < 0.01; ###, *p* < 0.001; ####, *p* < 0.0001; compared with the sham group. *, *p* < 0.05; **, *p* < 0.01; ***, *p* < 0.001; ****, *p* < 0.0001; compared with the PH group.

To investigate the effect of TUDCA on enhancing liver recovery post-hepatectomy, an animal study was conducted (Figure 2A). The findings indicated that liver regeneration was significantly greater in the TUDCA-treated group compared to the model group, as evidenced by an increase in the liver-to-body-weight ratio. This effect was more notable in the medium- and high-dose subgroups (Figure 2B). Additionally, serum analysis revealed that liver function markers, alanine aminotransferase (ALT) and aspartate aminotransferase (AST), were notably reduced in the treatment group compared to the model group. Conversely, the high-dose group showed a slight rise in these markers (Figure 2D,E). Histological analysis using hematoxylin and eosin (H&E) staining demonstrated a reduction in inflammation and edema in the treatment group relative to the model group (Figure 2C). Ki67 is the gold standard for cell proliferation and can specifically mark cells in an active proliferation cycle [18]. PCNA is a key factor in DNA replication and repair, and its expression level directly reflects the proliferation status of cells [19]. Expression levels of proliferation markers, including Ki67 and PCNA, were significantly increased in the TUDCA group, providing molecular evidence for its role in promoting liver regeneration (Figure 2C,F,G). Additionally, TUNEL staining revealed a noticeable reduction in apoptosis observed in the group receiving TUDCA, when compared to the control group. (Figure 2H,I). 

### 3.3. TUDCA Alleviates CCL_4_-Induced Liver Fibrosis

**Figure 3:**
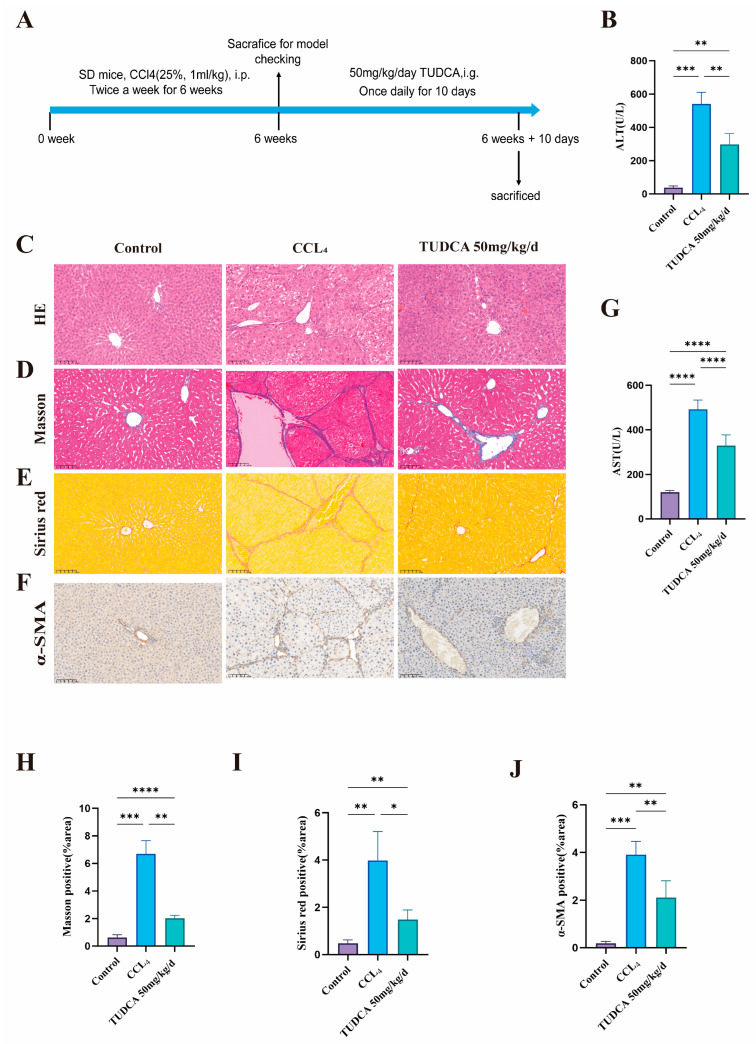
TUDCA Alleviates CCL_4_-Induced Liver Fibrosis. (**A**) Schematic diagram of the experimental design of TUDCA-treated CCL4-induced liver fibrotic rats. (n = 5). (**B**,**G**) ALT and AST levels. (**C**–**E**,**H**,**I**) Representative and statistical images of H&E (n = 3), Masson, and Sirius red staining. (scale bar, 100 μm). (**F**,**J**) Representative and statistical images of α-SMA staining. (scale bar, 100 μm) *, *p* < 0.05; **, *p* < 0.01; ***, *p* < 0.001; ****, *p* < 0.0001.

Liver fibrosis reduces hepatocyte proliferation and function, potentially leading to cirrhosis. Enhancing hepatocyte regeneration is a promising strategy to mitigate fibrosis. To assess TUDCA’s anti-fibrotic effects, we designed an animal experiment (Figure 3A). Based on the results of the liver regeneration study, medium-dose TUDCA was used. In the model group, ALT and AST levels, as well as H&E, Masson, and Sirius red staining, confirmed successful fibrosis induction. Compared to the model group, TUDCA treatment significantly reduced liver function markers (Figure 3B,G), improved cellular morphology (Figure 3C), and reduced fibrotic markers in Masson (Figure 3D,H) and Sirius red staining (Figure 3E,I). Moreover, TUDCA significantly decreased α-SMA expression, further supporting its anti-fibrotic effect (Figure 3F,J).

### 3.4. TUDCA Facilitates the Recovery of Liver Tissue Within a Fibrotic Environment Following Partial Liver Resection

**Figure 4:**
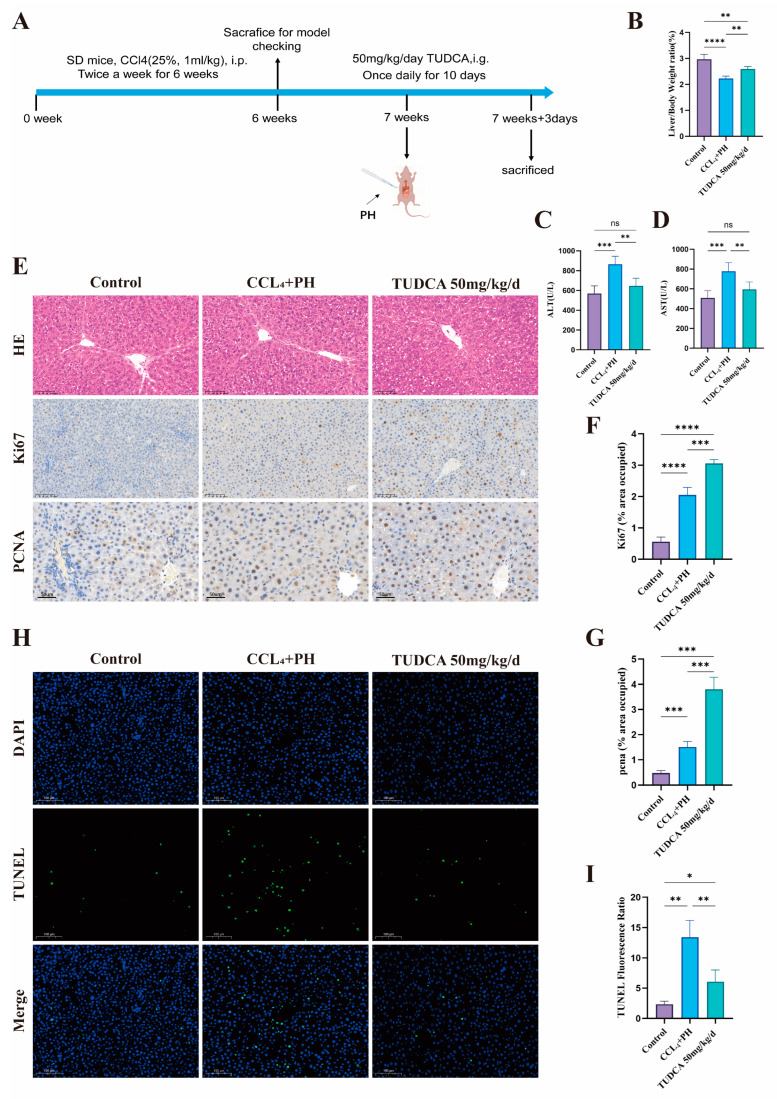
TUDCA facilitates the recovery of liver tissue within a fibrotic environment following partial liver resection. (**A**) Schematic of the experimental design for TUDCA treatment of 70% hepatectomised rats in the context of CCL_4_-induced fibrosis. (n = 5). (**B**) Liver weight ratio of rats treated according to A. (**C**,**D**) ALT and AST levels according to A-treated rats. (**E**–**G**) Representative and statistical images of H&E (n = 3) (scale bar, 100 μm), Ki67 (scale bar, 100 μm), and PCNA staining (scale bar, 50 μm). (**H**,**I**) TUNEL staining according to A-treated rats. (scale bar, 100 μm) n.s., not significant; *, *p* < 0.05; **, *p* < 0.01; ***, *p* < 0.001; ****, *p* < 0.0001.

To evaluate the influence of TUDCA on liver repair in fibrotic conditions, we conducted a study using a medium concentration of TUDCA (Figure 4A). Hepatic fibrosis was successfully induced using CCL_4_. Compared to the model group, the TUDCA treatment group demonstrated an increased liver-to-body weight ratio (Figure 4B) and significant reductions in ALT and AST levels, reflecting improved liver function (Figure 4C,D). H&E staining revealed decreased inflammation and edema (Figure 4E). Additionally, Ki67 and PCNA expression levels were significantly elevated (Figure 4E–G), while TUNEL staining showed reduced apoptosis in the TUDCA group (Figure 4H,I).

### 3.5. Mechanistic Study of TUDCA in Promoting Liver Proliferation

**Figure 5:**
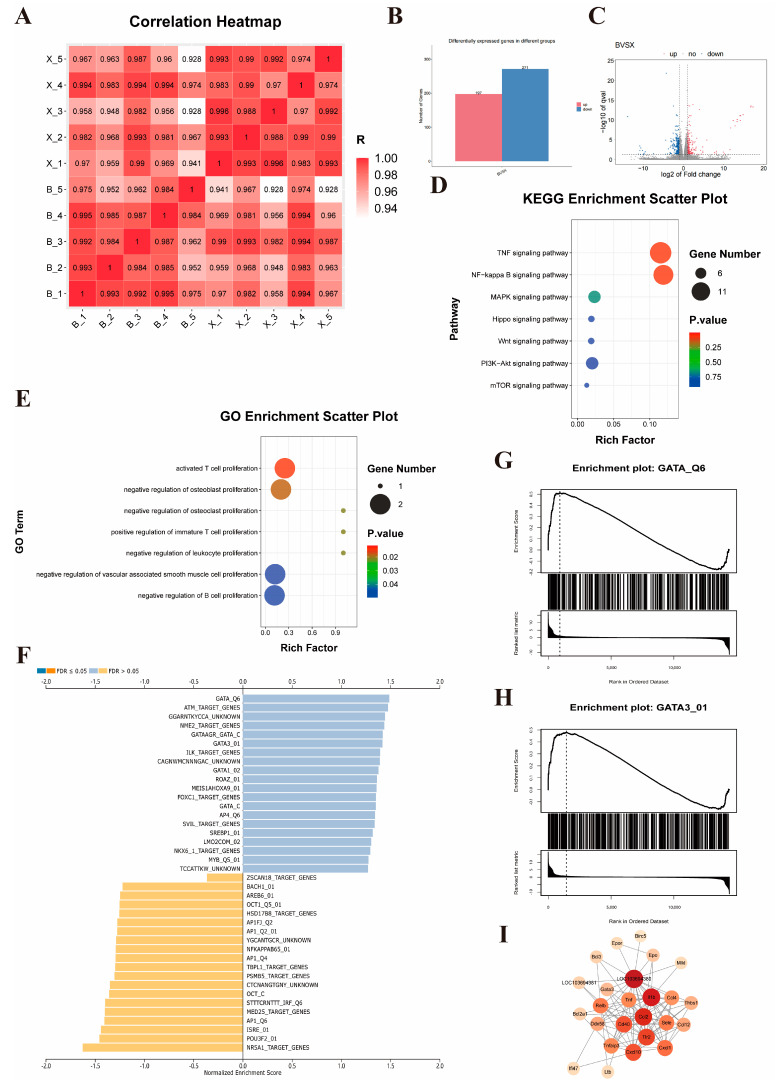
Mechanistic Study of TUDCA in Promoting Liver Proliferation (**A**) Heatmap of gene expression between PH_TUDCA and PH groups. (**B**) Differential gene expression between the two groups. (**C**) Volcano plot of differential gene expression between PH_TUDCA group and PH group.( Red indicates up-regulated genes and blue indicates down-regulated genes) (**D**) KEGG enrichment analysis of the differentially expressed genes between the PH_TUDCA group and the PH group. (**E**) GO enrichment analysis of the differentially expressed genes between the PH_TUDCA group and the PH group. (**F**) Transcription factor activity prediction based on differential gene profiles. (**G**,**H**) GSEA enrichment analysis of GATA and GATA3. (**I**) PPI analysis of GATA3.

To explore the mechanism by which TUDCA promotes the regeneration of the residual liver after partial hepatectomy (PH) in rats, we completed RNA-seq on the liver tissues of rats in the PH group and the PH_TUDCA group. The overall correlation between the two groups was good (Figure 5A), with 468 genes significantly altered in the PH_TUDCA group: 197 genes were upregulated, and 271 were downregulated (Figure 5B,C). Gene Ontology (GO) analysis of the genes with differential expression indicated that, compared with the PH group, the PH_TUDCA group exhibited enrichment in biological pathways, including the stimulation of T cell growth and the proliferation of immature T cells (Figure 5E). KEGG pathway enrichment analysis further identified upregulated pathways such as TNF signaling and NF-KappaB signaling in the PH_TUDCA group (Figure 5D), indicating a connection between TUDCA treatment and cell proliferation pathways.

However, the RNA-seq analysis results revealed hundreds of differentially expressed genes. This complex expression profile posed significant challenges for target identification and suggested that TUDCA might exert its biological effects through regulation of multiple signaling pathways and molecular networks. Faced with this challenge, we shifted to a more strategic research direction: identifying upstream transcription factors that might regulate these differentially expressed genes. This strategic shift was based on scientific considerations: first, transcription factors, as key transcriptional regulators, can simultaneously regulate the expression of hundreds of downstream genes [20]; second, transcription factor binding and regulation is a rapid and dynamic process, typically lasting only tens of seconds [21], meaning that relying solely on transcription factor expression levels in RNA-seq data might not accurately reflect their actual activity [22]. Based on this, we adopted an innovative strategy of reverse-predicting transcription factor activity through differential gene expression profiles, a method more accurate in reflecting their functional status than direct measurement of transcription factor expression levels [22,23]. This innovative approach identified GATA3 as the most significantly induced transcription factor by TUDCA (Figure 5F–H).

Next, we conducted a protein–protein interaction (PPI) analysis for GATA3 and its associated proliferation-related genes. The results showed regulatory relationships between GATA3 and genes such as Il1b, Tlr2, LOC103694380, Cd40, and Ccl2 (Figure 5I), strongly supporting the hypothesis that TUDCA mediates the proliferation process via GATA3.

### 3.6. Knockdown of GATA3 Abolishes TUDCA-Induced Proliferation in BRL-3A Cells

**Figure 6:**
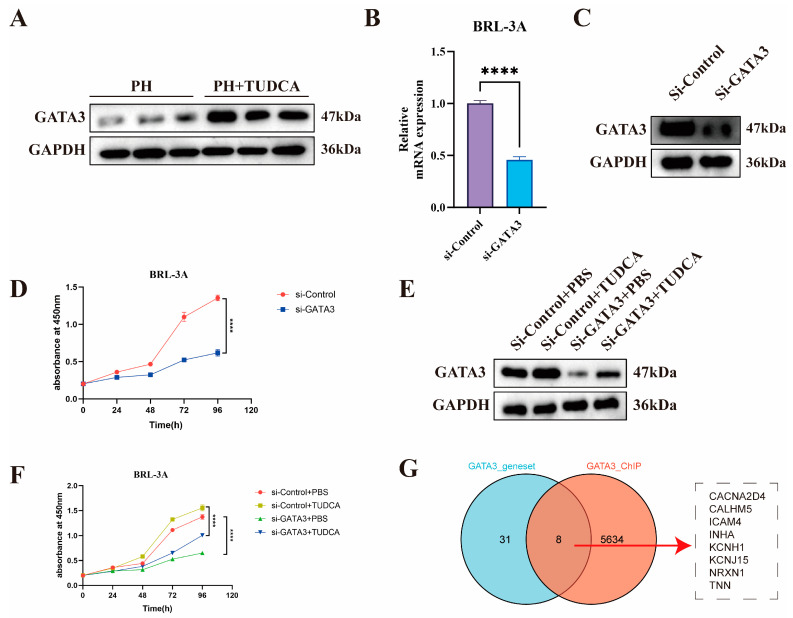
Knockdown of GATA3 Abolishes TUDCA-Induced Proliferation in BRL-3A Cells (**A**) GATA3 expression in liver tissues from PH and PH_TUDCA groups. (**B**,**C**) RT-qPCR and Western blot validation of GATA3 knockdown in BRL-3A cells. (**D**) Proliferation analysis of GATA3 knockdown cells. (**E**,**F**) Immunoblotting and proliferation analysis of BRL-3A cells treated with TUDCA or GATA3 knockdown. (**G**) Venn diagram illustrating the strategy for identifying GATA3 downstream target genes. ****, *p* < 0.0001.

To validate the mechanism by which TUDCA promotes liver regeneration, we designed the following experiments. GATA3 protein levels were significantly elevated in liver tissues of the PH_TUDCA group (Figure 6A). Using siRNA, we successfully knocked down *GATA3* in BRL-3A cells (Figure 6B,C), which resulted in a marked decrease in cell proliferation (Figure 6D). To further verify whether the effect of TUDCA on promoting liver regeneration depends on the activation of GATA3 gene, we knocked down GATA3 while treating TUDCA (Figure 6E). The experimental results clearly showed that although TUDCA can significantly promote cell proliferation, in GATA3 When the expression is inhibited, the pro-proliferation effect of TUDCA is significantly weakened (Figure 6F). This proves that TUDCA promotes liver regeneration by enhancing the expression and activity of GATA3.

To identify direct target gene networks regulated by the TUDCA-GATA3 signaling axis, we analyzed 39 potential GATA targets derived from differential expression analysis (GATA_Q6.csv). Additionally, ChIP-Seq data (GSM2817659) were annotated using ChIP seeker, revealing 7674 promoter-region binding sites, which were narrowed to 5642 after removing duplicates (peak_annotations.csv). The intersection of differential gene analysis and ChIP-Seq data yielded GATA3’s direct regulatory targets, visualized using a Venn diagram (Figure 6G). In addition, considering the differences between rats and humans, we used human datasets to increase the translatability of rat experimental results. Considering that the regulatory role of GATA3 in liver regeneration must involve stem cells, we used human trophoblast progenitors as the ChIP-Seq dataset.

## 4. Discussion

In our study, we systematically demonstrated the promoting effect of TUDCA on liver regeneration through a series of in vivo and in vitro experiments. In order to reveal the molecular mechanism of TUDCA promoting liver regeneration, we turned to a more strategic research direction based on the traditional differential gene analysis strategy: identifying upstream transcription factors that may regulate these differentially expressed genes. Surprisingly, this analysis method revealed that GATA3 was the most significant transcription factor induced by TUDCA. This finding is particularly intriguing because recent research published in Journal of Hepatology in 2024 (Wang et al.) suggests that GATA3 may have an inhibitory role in liver regeneration. In simple terms, reducing GATA3 expression while upregulating Ramp2 can promote liver sinusoidal endothelial cell proliferation, thereby negatively regulating intrahepatic vascular remodeling [24]. This apparent contradiction prompted us to deeply investigate the relationship between TUDCA and GATA3 and to reconsider the complex role of GATA3 in liver regeneration.

Through cell experiments, we found that GATA3 knockdown significantly inhibited hepatocyte proliferation. This result not only confirmed the importance of GATA3 in hepatocyte proliferation but also suggested that GATA3 might play different roles in different types of liver cells, with its function potentially depending on the specific stage of regeneration and cell type. This functional diversity and context dependency may explain the seemingly contradictory phenomena observed in previous studies. Such cell type-specific and context-dependent functional patterns of GATA3 are not uncommon in transcription factor research. A classic example is the role of β-catenin in different tissue development and regeneration. β-catenin shows distinctly different functions in hematopoietic stem cell development and skeletal formation: in hematopoietic stem cells, β-catenin activation inhibits their differentiation capacity [25], while in skeletal development, β-catenin activation promotes osteoblast differentiation and bone formation [26]. Similarly, GATA3 exhibits such tissue-specific actions: in T cell development, GATA3 plays a key role by promoting Th2 cell differentiation [27,28], while in kidney development, GATA3 plays an important role by regulating the differentiation of renal collecting duct epithelial cells [29,30]. These functional differences may arise from different cellular contexts, including different cofactor expression profiles, different signaling pathway networks, different epigenetic states, and different microenvironmental factors [31]. This understanding not only helps us comprehend the complexity of transcription factor function but also provides a theoretical basis for developing tissue-specific therapeutic strategies.

To further confirm whether TUDCA’s promotion of liver regeneration depends on GATA3, we designed a series of key validation experiments: knocking down GATA3 while treating with TUDCA. The experimental results clearly showed that although TUDCA could significantly promote cell proliferation, TUDCA’s pro-proliferative effect was markedly weakened when GATA3 expression was suppressed. This result directly demonstrated the necessity of GATA3 in TUDCA-promoted hepatocyte proliferation. More importantly, we also found that TUDCA could regulate GATA3 at multiple levels: not only upregulating its transcriptional activity but also increasing its mRNA and protein expression levels, which is consistent with previous literature reports. These findings further support the reliability and superiority of our strategy of reverse-predicting transcription factor activity through gene expression.

In conclusion, our research has for the first time elucidated the molecular mechanism by which TUDCA promotes liver regeneration: TUDCA promotes hepatocyte proliferation and liver regeneration by upregulating GATA3 expression and activity. This discovery not only reveals a new function of GATA3 in liver regeneration but also provides a new perspective for understanding TUDCA’s pharmacological effects. Our research also suggests that GATA3 may play a more complex role in liver regeneration than previously recognized, with its functions potentially varying significantly depending on cell type and regeneration stage.

To identify the direct target gene network of the TUDCA-GATA3 signaling axis, we employed a systematic research strategy combining bioinformatics analysis and experimental validation. We first utilized ChIP-Seq data from human embryonic stem cells (hESCs) in the ENCODE database [32] to analyze GATA3’s genomic binding site characteristics in detail, particularly focusing on its binding patterns in gene promoter regions. By integrating these binding site data with our transcriptome differential expression analysis results, we successfully identified a series of direct GATA3 target genes that likely play important roles in TUDCA-promoted liver regeneration.

These target genes include calcium channel-related proteins CACNA2D4 and CALHM5, cell adhesion molecule ICAM4, growth factor family member INHA, potassium channels KCNH1 and KCNJ15, nervous system-related protein NRXN1, and extracellular matrix protein TNN. The discovery of INHA as an important member of the TGF-β superfamily holds special significance [33,34]. INHA not only forms inhibin complexes with β subunits but also contains characteristic TGF-β family domains. Previous studies have confirmed its important role in FSH secretion regulation, cell proliferation, and differentiation processes. Considering the central position of TGF-β signaling in liver regeneration [35,36], particularly its role in regulating hepatocyte fate determination and extracellular matrix remodeling [37,38,39], the discovery of INHA provides a crucial molecular connection for understanding the GATA3 regulatory network.

Overall, our research findings form an interesting contrast with Wang et al.’s discoveries: they found that GATA3 shows inhibitory effects in intrahepatic vascular remodeling, while our results indicate it has promoting effects in hepatocytes. This apparent contradiction actually reveals the complexity of liver regeneration: the same transcription factor may play different or even opposite roles in different cell types and at different time points. This understanding not only deepens our comprehension of liver regeneration mechanisms but also provides important guidance for clinical treatment strategy development. These findings remind us that when developing and using targeted therapeutic drugs, we need to fully consider the differential effects of target genes in different cell types and at different time points.

While this study is the first to reveal a new mechanism by which TUDCA promotes hepatocyte proliferation through upregulating GATA3, several important research directions remain to be explored. Our work primarily focused on GATA3’s direct role in liver parenchymal cells (hepatocytes), confirming the crucial role of the TUDCA-GATA3 signaling axis in promoting hepatocyte proliferation. However, liver regeneration is a complex process where beyond autonomous hepatocyte proliferation, immune microenvironment regulation is equally indispensable. Considering GATA3’s central position in the immune system, particularly its key role in T cell differentiation and inflammatory response regulation, we have reason to believe that the TUDCA-GATA3 signaling axis might influence liver regeneration through modulating the immune microenvironment. For instance, as the main regulator of Th2 responses, GATA3 might coordinate the regeneration process by influencing the local inflammatory environment [27]. Several GATA3 target genes we identified (such as ICAM4) might also participate in immune cell recruitment and activation [40,41]. This suggests that TUDCA’s therapeutic effects might be partially achieved through immune response regulation. Therefore, future research directions could include: (1) Studying the effects of the TUDCA-GATA3 signaling axis on liver immune cell composition; (2) exploring GATA3’s expression and function in different intrahepatic immune cell subpopulations; (3) analyzing changes in the local inflammatory environment after TUDCA treatment; and (4) evaluating the role of immune regulation in TUDCA-promoted liver regeneration. These studies will help us better understand TUDCA’s therapeutic mechanisms comprehensively and might provide new insights for developing more effective treatment strategies. Our findings reveal that GATA3 might play different or even opposite roles in different cell types, and this spatiotemporal specificity suggests that we need to consider treatment time windows and cell type specificity in clinical applications. This research will help us develop a more comprehensive understanding of TUDCA’s therapeutic mechanisms and provide a theoretical foundation for developing more precise treatment strategies. In particular, our discovery that GATA3 may play different or even opposite roles in different cell types suggests that we need to consider treatment time windows and cell-type specificity in clinical applications.

## Data Availability

The datasets associated with this study can be accessed through online repositories. Raw RNA sequencing data are available upon reasonable request.

## Supplementary Materials

The following supporting information can be downloaded at: https://www.mdpi.com/article/10.3390/biomedicines13040910/s1, Table S1: Primers and SIRNA sequences; Table S2: GATA_Q6; Table S3: peak_annotations.
