# Supplementary material for: Tauroursodeoxycholic Acid Induces Liver Regeneration and Alleviates Fibrosis Through GATA3 Activation
Source: Biomedicines. 2025 Apr 9;13(4):910. doi: 10.3390/biomedicines13040910 (PMC12024728; doi:10.3390/biomedicines13040910)
Supplement: Supplementary file 1 [file biomedicines-13-00910-s001.zip › Table S1-Primers and SIRNA sequences.pdf]

Table. The specific primer sequences used in the qRT-PCR analysis in this study

Target	Sequences (5′-3′)
GATA3	Forward: CTCTTCCCTCCCAGCAGCCTAC
	Reverse: AGTACCATCTCGCCGCCACAG
GAPDH	Forward: CAAGTTCAACGGCACAGTCAAGG
	Reverse: ACATACTCAGCACCAGCATCACC

Table. The Si-RNA sequences used in this study

Target	Sequences (5′-3′)
rGATA3-760	Sense: GGCACGAUCCAGCACAGAA/dT//dT/
	Antisense: UUCUGUGCUGGAUCGUGCC/dT//dT/
